# Attitude and Heading Estimation for Indoor Positioning Based on the Adaptive Cubature Kalman Filter

**DOI:** 10.3390/mi12010079

**Published:** 2021-01-13

**Authors:** Jijun Geng, Linyuan Xia, Dongjin Wu

**Affiliations:** 1 Guangdong Provincial Key Laboratory of Urbanization and Geo-Simulation, School of Geography and Planning, Sun Yat-sen University, 135 # Xingangxi Road, Guangzhou 510275, China; gengjj@mail2.sysu.edu.cn; 2 School of Geomatics Science and Technology, Nanjing Tech University, 30# Puzhu Road, Nanjing 211816, China

**Keywords:** indoor positioning, MARG sensors, attitude and heading, adaptive cubature Kalman filter

## Abstract

The demands for indoor positioning in location-based services (LBS) and applications grow rapidly. It is beneficial for indoor positioning to combine attitude and heading information. Accurate attitude and heading estimation based on magnetic, angular rate, and gravity (MARG) sensors of micro-electro-mechanical systems (MEMS) has received increasing attention due to its high availability and independence. This paper proposes a quaternion-based adaptive cubature Kalman filter (ACKF) algorithm to estimate the attitude and heading based on smart phone-embedded MARG sensors. In this algorithm, the fading memory weighted method and the limited memory weighted method are used to adaptively correct the statistical characteristics of the nonlinear system and reduce the estimation bias of the filter. The latest step data is used as the memory window data of the limited memory weighted method. Moreover, for restraining the divergence, the filter innovation sequence is used to rectify the noise covariance measurements and system. Besides, an adaptive factor based on prediction residual construction is used to overcome the filter model error and the influence of abnormal disturbance. In the static test, compared with the Sage-Husa cubature Kalman filter (SHCKF), cubature Kalman filter (CKF), and extended Kalman filter (EKF), the mean absolute errors (MAE) of the heading pitch and roll calculated by the proposed algorithm decreased by 4–18%, 14–29%, and 61–77% respectively. In the dynamic test, compared with the above three filters, the MAE of the heading reduced by 1–8%, 2–18%, and 2–21%, and the mean of location errors decreased by 9–22%, 19–31%, and 32–54% respectively by using the proposed algorithm for three participants. Generally, the proposed algorithm can effectively improve the accuracy of heading. Moreover, it can also improve the accuracy of attitude under quasistatic conditions.

## 1. Introduction

Location-based services (LBS) are becoming more and more popular because of the exponential use of mobile devices [1]. As we know, location is an essential part of LBS. Global navigation satellite system (GNSS), including GPS, GLONASS, Galileo, Beidou navigation satellite system (BDS) and other regional systems, provide accurate location services outdoors. However, due to the limitations of satellite signals, GNSS is always unavailable in indoor environments. Thus, extra sensors are necessary to strengthen indoor positioning. In the past decades, WiFi [2,3,4], Bluetooth [5,6,7], ultra-wideband (UWB) [8,9,10], and micro-electro-mechanical system (MEMS) [11,12,13] have been studied for indoor positioning. Among these techniques, MEMS sensors are more competitive as their independence of the existing infrastructures in indoor environments [14,15,16]. Moreover, MEMS magnetic, angular rate, and gravity (MARG) sensors are lightweight, low-cost, and more and more accurate, which greatly facilitates their application in indoor positioning [17]. Numerous studies, in terms of pedestrian dead reckoning (PDR) [18,19,20], intelligent robots [20,21,22], and indoor UAV navigation [23] are dedicated to estimating the attitude and heading based on MEMS MARG sensors. Currently, MARG sensors are embedded in each smartphone. Thus the attitude and heading of a smartphone are conveniently estimated using data from the MARG sensors. Attitude and heading are important for motion tracking applications and indoor positioning and navigation. Real-time position of a pedestrian can be easily obtained from the smartphone information provided that relative attitude and heading between the pedestrian and his/her smartphone. In addition, the attitude information from the MARG sensors potentially improves the heading estimation, because it can further consider the effects of the roll and pitch [24].

Attitude and heading is usually represented by the Euler angle, which consists of roll, pitch, and yaw angles. Roll and pitch angles can be used to represent the attitude, and heading can be computed from the yaw angle. In quasistatic conditions or a magnetically clean environment, the attitude and heading can be calculated based on the measured gravitational acceleration and the geomagnetic field. However, the values are easily impacted by the surrounding environment or other factors, which cause them to fluctuate greatly around the true value. At the same time, the attitude and heading can also be derived from the gyro angular rate. However, due to the integration process in the inertial navigation mechanization, the gyro sensor error will accumulate, resulting in unreliable estimation, especially in the case of a long time running. Naturally, the attitude and heading should be calculated by fusing the aforementioned sensor data, and the fusion is always employing a complementary filter (CF) and Kalman filter (KF) [25].

CF is relatively easy to implement and requires low computational cost. However, the accuracy of attitude and heading produced by CF is slightly lower than that estimated by KF, and even worse in dynamic environments [25]. The Kalman filter utilizes raw measurement from the receiver to estimate the system state [26]. The KF assumes that the system model is known and linear, and the system noise and measurement noise are white. If these assumptions are violated, the KF’s result will be suboptimal and unstable. To solve the nonlinear problem, the extended Kalman filter (EKF) [27] algorithm is proposed. Although the EKF model is linearized by first-order Taylor series expansion of the process/measurement models for the current state estimate, it is difficult to obtain the ideal estimation, when the system has strong nonlinearity or the initial estimation error is large. In the Gaussian case, sigma point approaches used in an unscented Kalman filter (UKF) [28] and cubature Kalman filter (CKF) [29] have appeared. Compared with EKF, UKF and CKF provide estimates with higher accuracy and avoid the calculation of the Jacobian matrix. However, the UKF does not have a strict mathematical derivation. In the filter iteration process, state estimation covariance is difficult to maintain being positive due to matrix decomposition and inversion. Especially when the state dimension is high, the UKF filter accuracy will diverge. CKF was proposed by Arasaratnam and Haykin [30]. The core is to capture the statistical characteristics of random variables after the nonlinear transformation according to basic cubature points generated by the spherical-radial cubature rule [31]. Compared with EKF, CKF does not need linearization of the nonlinear model and calculation of the Jacobian matrix. Moreover, compared with UKF, CKF has stronger adaptability, especially for high-dimensional estimation [31,32]. Therefore, CKF is seen as a powerful tool to solve the nonlinear estimation problem in the data fusion of MARG sensors.

However, a priori statistical properties of the noise should be accurately known in the design of the CKF algorithm. On the one hand, due to the restriction of testing samples, the statistical apriority is hard to know exactly in practical application. Furthermore, in the actual operational environment, the statistical characteristics become complex and change dynamically because of various uncertain factors [32]. Uncertainties of noise statistics and the system model make the accuracy of estimation decrease or even diverge. In order to solve the problem, there have been many efforts undertaken to develop an adaptive CKF to enhance the adaptability and robustness [31,32,33,34,35,36,37]. The adaptive filter algorithms, using some types of statistic estimator to evaluate the unknown or time-varying noise, including the Sage-Husa estimator [31,32], variational Bayesian [33,34], maximum likelihood [35,36], maximum a posteriori [37], and covariance matching [38]. Therein, the Sage-Husa estimator is one of the widely used adaptive methods, since it has the advantages that the recursive formula is simple and the principle is clear and easy to implement [31,32]. In the traditional Sage-Husa cubature Kalman filter (SHCKF), the noise parameters are estimated by the equal-weighted time-averaging algorithm. When the noise parameter is a known fixed value, and the estimation is effective and convergent, and the estimation of the noise parameter is more and more accurate with the increase of time. However, MARG sensors of smartphones have the characteristics of low cost and low accuracy, and their noise characteristics are easily changed by the external environment. Therefore, the parameters of the MARG sensors’ noise updated in real-time can better reflect the current data characteristics.

When the noise statistical characteristics change rapidly, recent observation data plays an important role in estimating the noise statistics at the current time, while too old data have little effect. Especially for a complex motion process, the true value of noise statistics at the current moment has a stronger correlation with recent historical data. Another problem is that if the Kalman gain becomes smaller and smaller, the role of the new measurement data reflecting the true state to a certain extent is getting weaker and weaker in the estimation, which results in data saturation and may cause the filter to diverge. In some cases, especially when the order of the system is relatively high, there is often a divergence of values. Besides, dynamic model errors are difficult to accurately describe in advance, and for sudden steering, large dynamic model errors are introduced into the filter [39]. In response to these problems, this paper proposes a quaternion-based adaptive cubature Kalman filter (ACKF) to estimate the attitude and heading of a smartphone with its embedded sensors’ data. To adaptively correct the noise parameters of the nonlinear system and reduce the estimation bias of the filter, the fading memory weighted method and the limited memory weighted method are used to replace the equal-weighted time-averaging algorithm to estimate and correct the model noise parameters. Then the statistical properties of the nonlinear system are estimated and corrected by the fading memory weighted method and limited memory weighted method. At the same time, according to the characteristics of pedestrian walking, this paper proposes that the latest step data is used as the memory window data of the limited memory weighted method in this paper. The accuracy of the filter estimation is enhanced by increasing the weight of the latest historical observation data and reducing the old data weights. Moreover, based on the stability of the filter, the paper analyzes the reasons for the divergence of the filter algorithm, and the filter innovation sequence is used to rectify the measurement and system noise covariance for restraining the divergence. Besides, to balance the contributions of the measurements and the dynamic model information, an adaptive factor based on prediction residual construction is used to overcome the filter model error and the influence of abnormal disturbance.

Theoretical analysis and experimental results show that the proposed ACKF algorithm can provide better accuracy, and effectively eliminate interference from dynamic noise compared with conventional SHCKF, CKF and EKF. In general, the contributions of our work can be summarized as follows. First of all, an adaptive cubature Kalman filter algorithm combining the fading memory factor and the limiting memory factor is proposed to estimate the attitude and heading based on data from MARG sensors. According to the characteristics of pedestrian walking, the latest step data is used as a memory window in the pedestrian walking process. Second, by judging the positive definiteness of the measurement and system noise covariance matrix, the possible filter divergence is suppressed by correcting the measurement and system noise covariance. Finally, the paper uses an adaptive factor to weaken the influence of the filter model errors and abnormal disturbances. In addition, static and dynamic experiments were conducted. In terms of the heading, the proposed algorithm can provide a more stable and accurate heading estimation information. While for the attitude, the proposed algorithm can effectively improve the accuracy under quasistatic conditions.

The rest of this paper is organized as follows: In Section 2 the attitude and heading estimations for indoor positioning are described in detail. In Section 3 we explain the proposed method in general. Experiments and results analysis are given in Section 4. Discussion is given in Section 5. Finally, the conclusions and future work are presented in Section 6.

## 2. Methodology

**Figure 1:**
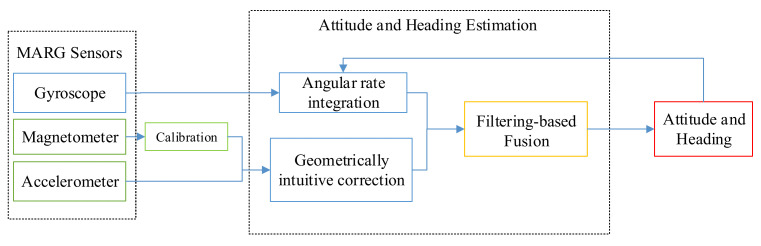
The frame of the fusion filter algorithm [25].

Low-cost MARG sensors embedded in the smartphone, such as an accelerometer, magnetometer, and gyroscope provide raw data for attitude and heading estimation [25]. As Figure 1 presents, the outputs from the accelerometer and magnetometer can be used to calculate the attitude and heading. Moreover, the attitude and heading can also be computed by the angular rate. The two kinds of calculations are then fused using a filter algorithm, and the optimal values are produced iteratively.

**Figure 2:**
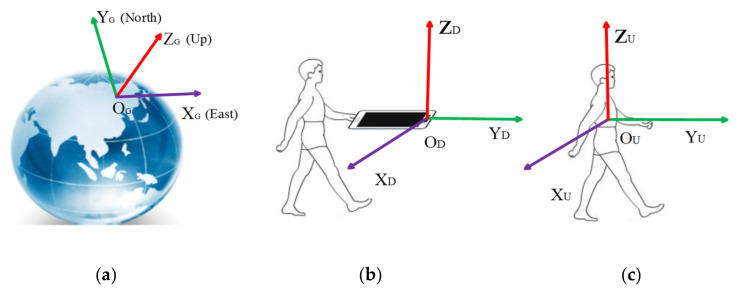
Illustration of the defined coordinate systems, (**a**) the local Cartesian coordinates coordinate system, (**b**) the device coordinate system, (**c**) the user coordinate system.

The attitude and heading for a smartphone are defined as the relative orientation of its device coordinate system concerning a reference coordinate system [40]. To explain the attitude and heading, we defined three different coordinate systems, as depicted in Figure 2. The local Cartesian coordinates coordinate system is defined that the X_G_, Y_G_, and Z_G_ axes point east, north, and sky, respectively in Figure 2a. MARG sensors data outputted by built-in sensors of a smartphone is always organized depending on the device coordinate system, as shown in Figure 2b. X_D_ and Y_D_ axes are on the same plane with the phone screen pointing rightward and forward respectively, and the Z_D_ axis points out of the phone screen under the right-hand rule. For indoor positioning, the attitude and heading values of the smartphone are further transformed to derive pedestrian heading. Additionally, therefore, a user coordinate system is necessary. We can see from Figure 2c, Y_U_ axis points forward aligning with the orientation of the user’s body. Z_U_ axis is coinciding with Z_G_. X_U_ axis is the right side of the user body and obtained by the cross product of Y_U_ and Z_U_.

### 2.1. Attitude and Heading in the Form of a Quaternion

Attitude and heading can be expressed using several parameterizations, such as the Euler angles, Rodrigues parameters, the quaternion, etc. Quaternion has been widely used because of its less computation burden and global non-singularity. A quaternion consists of four elements:

(1) $$q\left(q_{0}\;q_{1}\;q_{2}\;q_{3}\right)=q_{0}+q_{1}i+q_{2}j+q_{3}k$$

where $q_{0}$, $q_{1}$, $q_{2}$, and $q_{3}$ are real numbers, and **i**, **j,** and **k** are unit vectors. The quaternion satisfies the constraint of the unit norm.

(2) $$q_{0}^{2}+q_{1}^{2}+q_{2}^{2}+q_{3}^{2}=1$$

Let $q_{0}=cos\frac{\theta }{2}$, $q_{1}=lsin\frac{\theta }{2}$, $q_{2}=msin\frac{\theta }{2}$, $q_{3}=nsin\frac{\theta }{2}$, the quaternion equation is:

(3) $$q\left(q_{0},q_{1},q_{2},q_{3}\right)=cos\frac{\theta }{2}+(li+mj+nk)sin\frac{\theta }{2}=cos\frac{\theta }{2}+u^{R}sin\frac{\theta }{2}$$

where θ is the rotation angle and **u** is a unit vector.

When the body coordinate system is **b** (in this paper, the body coordinate system is the device coordinate system), and the navigation coordinate system is **n**, $C_{b}^{n}$ is the coordinate transformation matrix from the **b** coordinate system to the **n** coordinate system, which can be used to calculate the heading angle and attitude angle. Then coordinate transformation matrix $C_{b}^{n}$ can be described as [41]:

(4) $$C_{b}^{n}=I+2Usin\frac{\theta }{2}cos\frac{\theta }{2}+2{sin}^{2}\frac{\theta }{2}U\times U$$

where $U=\left(\begin{array}{ccc} 0 & -n & m\\ n & 0 & -l\\ -m & l & 0 \end{array}\right)$.

Combining $q_{0}=cos\frac{\theta }{2}$, $q_{1}=lsin\frac{\theta }{2}$, $q_{2}=msin\frac{\theta }{2}$, and $q_{3}=nsin\frac{\theta }{2}$, the Equation (4) can be expressed as the following:

(5) $$C_{b}^{n}=\left[\begin{array}{ccc} 1-2\left(q_{2}^{2}+q_{3}^{2}\right) & 2\left(q_{1}q_{2}-q_{0}q_{3}\right) & 2\left(q_{1}q_{3}+q_{0}q_{2}\right)\\ 2\left(q_{1}q_{2}+q_{0}q_{3}\right) & 1-2\left(q_{1}^{2}+q_{3}^{2}\right) & 2\left(q_{2}q_{3}-q_{0}q_{1}\right)\\ 2\left(q_{1}q_{3}-q_{0}q_{2}\right) & 2\left(q_{2}q_{3}+q_{0}q_{1}\right) & 1-2\left(q_{1}^{2}+q_{2}^{2}\right) \end{array}\right]$$

Moreover, in the navigation coordinate system, the directions of X_G_, Y_G_, and Z_G_ are specified as East, North, and Up. A coordinate transformation matrix corresponding to three basic rotations can be expressed as:

$$C_{\psi }^{Z}=\left[\begin{array}{ccc} cos\psi & -sin\psi & 0\\ sin\psi & cos\psi & 0\\ 0 & 0 & 1 \end{array}\right],C_{\varphi }^{Y}=\left[\begin{array}{ccc} cos\varphi & 0 & -sin\varphi \\ 0 & 1 & 0\\ sin\varphi & 0 & cos\varphi \end{array}\right],C_{\theta }^{X}=\left[\begin{array}{ccc} 1 & 0 & 0\\ 0 & cos\theta & sin\theta \\ 0 & -sin\theta & cos\theta \end{array}\right]$$

where ψ is the yaw angle; θ is the pitch angle; and φ is the roll angle.

Then the attitude matrix between the body coordinate system **b** and the navigation coordinate system **n** is shown in the following:

(6) $$\begin{array}{cc} C_{n}^{b} & =C_{\varphi }^{Y}C_{\theta }^{X}C_{\psi }^{Z}\\ & =\left[\begin{array}{ccc} cos\varphi cos\psi +sin\varphi sin\psi sin\theta & -cos\varphi sin\psi +sin\varphi cos\psi sin\theta & -sin\varphi cos\theta \\ sin\psi cos\theta & cos\psi cos\theta & sin\theta \\ sin\varphi cos\psi -cos\varphi sin\psi sin\theta & -sin\varphi sin\psi -cos\varphi cos\psi sin\theta & cos\varphi cos\theta \end{array}\right] \end{array}$$

The coordinate system is always a rectangular coordinate system during the rotation from the **n** coordinate system to the **b** coordinate system. So, $C_{n}^{b}$ is the orthogonal matrix and $C_{n}^{b}={\left(C_{b}^{n}\right)}^{T}$. Combining Equations (5) and (6), the Euler angle can be described as:

(7) $$\begin{array}{c} \theta =arcsin\left[2\left(q_{2}q_{3}+q_{0}q_{1}\right)\right]\\ \varphi =arctan\left[-\frac{2\left(q_{1}q_{3}-q_{0}q_{2}\right)}{1-2\left(q_{1}^{2}+q_{2}^{2}\right)}\right]\\ \psi_{m}=arctan\left[\frac{2\left(q_{1}q_{2}-q_{0}q_{3}\right)}{1-2\left(q_{1}^{2}+q_{3}^{2}\right)}\right] \end{array}$$

The heading can be determined with the ψ.

(8) $$\psi =\psi_{m}+D$$

where D is the local declination angle.

### 2.2. Attitude and Heading Estimation with Readings of a Gyroscope

The attitude and heading can be computed according to initial values and angular rates outputted by a gyroscope. The quaternion $\dot{q}$, representing the changed attitude and heading from the previous quaternion, can be expressed as:

(9) $$\dot{q}=\frac{1}{2}q⊗w$$

where **w** is the angular rate vector with its quaternion form [0, $w_{x}$, $w_{y}$, $w_{z}$]^T^, in which $w_{x}$, $w_{y}$, $w_{z}$ are angular rate values along *x*, *y* and *z* axes of the device coordinate system.

The matrix form of (9) can be written as:

(10) $$\dot{q}=\frac{1}{2}M\left(w\right)q=\frac{1}{2}\left[\begin{array}{cccc} 0 & -w_{x} & -w_{y} & -w_{z}\\ w_{x} & 0 & w_{z} & -w_{y}\\ w_{y} & a_{32} & 0 & w_{x}\\ w_{z} & w_{y} & -w_{x} & 0 \end{array}\right]\left[\begin{array}{c} q_{0}\\ q_{1}\\ q_{2}\\ q_{3} \end{array}\right]$$

According to the Peano-Baker series, the solution of (10) is:

(11) $$q_{k+1}=[I+\frac{\Delta t}{2}\Delta \Theta ]q_{k}$$

where **I** is the n*n unit matrix, Δt is the sampling interval, ∆Θ is the incremental angle matrix with its form of $\Theta_{x}$, $\Theta_{y}$, and$\Theta_{z}$.

According to Equations (7) and (11), an estimate of the pitch, roll, and heading can be obtained based on the angular rate.

### 2.3. Attitude and Heading Estimation with Readings of an Accelerometer and a Magnetometer

Pitch and roll angles can be achieved by the accelerometer information too. According to the definition of the global coordinate system and device coordinate system in the above, the relationship between gravitational acceleration components in the frame **b** and the gravity vector in the frame **n**, when regardless of its acceleration of the multisensory system, can be written as [14]:

(12) $$\left[\begin{array}{c} g_{x}\\ g_{y}\\ g_{z} \end{array}\right]=C_{n}^{b}\left[\begin{array}{c} 0\\ 0\\ g \end{array}\right]=\left[\begin{array}{c} -gcos\theta sin\varphi \\ gsin\theta \\ gcos\theta cos\varphi \end{array}\right]$$

where $g_{x}$, $g_{y}$, and $g_{z}$ denote the measurements of the accelerometer, and g represents the local gravitational acceleration. Then, the pitch and roll can be obtained as follows:

(13) $$\left\{\begin{array}{c} \theta =arcsin\left(\frac{g_{y}}{g}\right)\\ \varphi =arctan\left(-\frac{g_{x}}{g_{z}}\right) \end{array}\right.$$

In addition, the measured magnetic field of the magnetometer can be used to compute the yaw angle. Before that, the magnetometer often needs to be calibrated to eliminate the impacts of hard iron, soft iron, and scale factor [25]. In this paper, we tried to cut down the effect of the hard iron and scale factor. The magnetic field correction model can be established as the following. Detailed implementation of the model refers to [25]:

(14) $$m=K\left(m^{*}+m_{0}\right)=diag\left(K_{x},K_{y},K_{z}\right)(\left[\begin{array}{c} m_{x}^{*}\\ m_{y}^{*}\\ m_{z}^{*} \end{array}\right]+\left[\begin{array}{c} m_{x0}\\ m_{y0}\\ m_{z0} \end{array}\right])$$

where $m={\left[m_{x}\;m_{y}\;m_{z}\right]}^{T}$, **K** denotes a scale transformation matrix.

A standard three-axis magnetometer reads the magnetic field in an aircraft’s body axis system as ($m_{x}$, $m_{y}$, and $m_{z}$). The relationship between magnetometer readings and the Earth’s magnetic field vector ($m_{E}$, $m_{N}$, and $m_{S}$) arranged in East, North, and Sky is as follows:

(15) $$\left[\begin{array}{c} m_{x}\\ m_{y}\\ m_{z} \end{array}\right]=(C_{n}^{b})\left[\begin{array}{c} m_{E}\\ m_{N}\\ \;m_{S} \end{array}\right]={\left(C_{b}^{n}\right)}^{T}\left[\begin{array}{c} m_{E}\\ m_{N}\\ m_{S} \end{array}\right]$$

where $C_{b}^{n}$ is the transformation matrix between the body coordinate system and the navigation coordinate system.

To determine the yaw angles, the geomagnetic vector can be resolved onto a local tangent plane. Hence, Equation (15) can be rearranged as follows [42]:

(16) $$\left[\begin{array}{c} m_{h}sin\psi \\ m_{h}cos\psi \\ m_{S} \end{array}\right]=\left(\begin{array}{ccc} cos\varphi & 0 & sin\varphi \\ sin\varphi sin\theta & cos\theta & -cos\varphi sin\theta \\ -sin\varphi cos\theta & sin\theta & cos\varphi cos\theta \end{array}\right)\left[\begin{array}{c} m_{x}\\ m_{y}\\ m_{z} \end{array}\right]$$

where $m_{h}sin\psi$ and $m_{h}cos\psi$ are the *x*- and *y*-axis components in the h-frame, respectively.

According to Equation (16), the heading angle can be derived as follows:

(17) $$\psi =arctan\left(\frac{m_{x}cos\varphi +m_{z}sin\varphi }{m_{x}sin\theta sin\varphi +m_{y}cos\theta -m_{z}cos\theta sin\varphi }\right)+D$$

Taking into account the local magnetic declination, the actual local heading based on the magnetometer was computed.

Finally, according to Equations (13) and (17), the attitude and heading were obtained.

The above two methods for attitude and heading estimation could be fused to achieve more accurate results, and a frame of adaptive cubature Kalman filter was applied in this paper. In the following, the process of adaptive cubature Kalman filter algorithm for attitude and heading estimation was explained in detail.

## 3. Quaternion-Based Adaptive Cubature Kalman Filter Algorithm for Attitude and Heading Estimation

**Figure 3:**
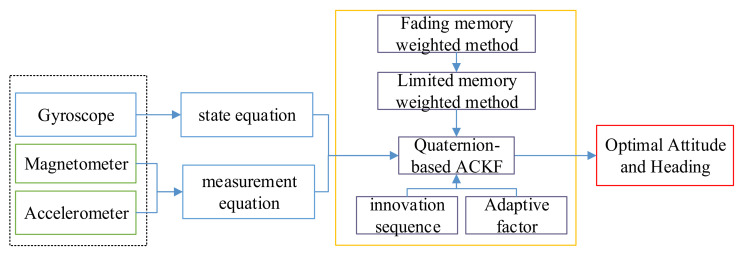
The frame of the quaternion-based adaptive cubature Kalman filter (ACKF) algorithm.

A quaternion-based adaptive cubature Kalman filter algorithm was proposed to estimate the attitude and heading, and its frame is shown in the figure below. Compared with EKF, ACKF does not need linearization of the nonlinear model and calculation of the Jacobian matrix, and ACKF has stronger adaptability compared with UKF. Therefore, the ACKF can be used for estimating the attitude and heading through fusing the outputs of the accelerometer, magnetometer, and gyroscope. To weaken the influence of system model errors and measurement outliers, the fading memory weighted and limited memory weighted methods were applied. Moreover, the filter innovation sequence was used to rectify the measurement noise covariance and system noise covariance matrices for restraining the divergence. Besides, an adaptive factor based on prediction residual construction was used to overcome the filter model error and the influence of abnormal disturbance, as shown in Figure 3.

### 3.1. Measuring and State Model

The two kinds of attitude and heading estimates will be designed to form the states and measurements in the filter algorithm. Denote $X_{k}$ = ${\left[{\hat{q}}_{0}\;{\hat{q}}_{1}\;{\hat{q}}_{2}\;{\hat{q}}_{3}\right]}^{T}$ as the state at time k, $z_{k}$ = ${\left[\theta \;\varphi \;\psi \right]}^{T}$ as the measurement at time k. The state equation is written as:

(18) $$X_{k}={\left[\begin{array}{l} {\hat{q}}_{0}\\ {\hat{q}}_{1}\\ {\hat{q}}_{2}\\ {\hat{q}}_{3} \end{array}\right]}_{k}=\frac{\Delta t}{2}\left(\begin{array}{cccc} 1 & -w_{x} & -w_{y} & -w_{z}\\ w_{x} & 1 & w_{z} & -w_{y}\\ w_{y} & -w_{z} & 1 & w_{x}\\ w_{z} & w_{y} & -w_{x} & 1 \end{array}\right){\left[\begin{array}{l} q_{0}\\ q_{1}\\ q_{2}\\ q_{3} \end{array}\right]}_{k-1}+w_{k-1}$$

where $w_{k-1}$ denotes the model noise. The measurement equation is written as:

(19) $$z_{k}={\left[\theta \;\varphi \;\psi \right]}^{T}=\left[\begin{array}{c} arcsin\left[2\left(q_{2}q_{3}+q_{0}q_{1}\right)\right]\\ arctan\left[-\frac{2\left(q_{1}q_{3}-q_{0}q_{2}\right)}{1-2\left(q_{1}^{2}+q_{2}^{2}\right)}\right]\\ arctan\left[\frac{2\left(q_{1}q_{2}-q_{0}q_{3}\right)}{1-2\left(q_{1}^{2}+q_{3}^{2}\right)}\right] \end{array}\right]+v_{k}$$

where $v_{k}$ denotes the measurement noise.

According to Equations (18) and (19), the filter considers the following process and observation models:

(20) $$\left\{\begin{array}{c} X_{k}=F_{k-1}X_{k-1}+w_{k-1}\\ z_{k}=h\left(X_{k}\right)+v_{k} \end{array}\right.$$

where $X_{k}$ and $z_{k}$ represent the system state and measurement at time instant k; h (.) is known vector mappings. $w_{k-1}$ and $v_{k}$ are the noise of the process and measurement. Nonlinear filter estimates unknown system states based on the current time and previous noisy observations.

### 3.2. Conventional Cubature Kalman Filter Algorithm

#### 3.2.1. Cubature Rule

Since the mean and variance can express the Gaussian distribution, the Gaussian filter of the Kalman filter structure can be used to process the state estimation task. The general form is as follows:

(21) $$\left\{\begin{array}{c} {\hat{x}}_{k|k}={\hat{x}}_{k|k-1}+W_{k}(z_{k}-{\hat{z}}_{k})\\ P_{k|k}=P_{k|k-1}-W_{k}P_{zz,k|k-1}W_{k}^{T}\\ W_{k}=P_{xz,k|k-1}{P_{zz,k|k-1}}^{-1} \end{array}\right.$$

where ${\hat{x}}_{k|k}$ and $P_{k|k}$ are the mean and variance of a probability distribution $p(x_{k}|Z_{k})$. ${\hat{x}}_{k|k-1}$ and $P_{k|k-1}$ are the state prediction value and its covariance at k time, ${\hat{z}}_{k}$ and $P_{zz,k|k-1}$ are predicted measurement and covariance. $P_{xz,k|k-1}$ is the predicted cross-covariance. $W_{k}$ is Kalman gain.

The calculation of the above mathematical expectations involves the same dimension as the system state. Consider a multidimensional weighted integral of the form:

(22) $$I\left(T\right)=\int_{D}T\left(x\right)w\left(x\right)d\left(x\right)$$

where $T\left(.\right)$ is some arbitrary function, $D\subseteq R^{n}$ is the region of integration, and the known weighted function w(x) ≥ 0.

In general, the solution to the above equation is difficult to obtain. So, it is necessary to find numerical integration methods to compute it. Based on the spherical-radial cubature rule, the cubature Kalman filter can be used to calculate Equation (22). The basic task for computing the equation by spherical-radial cubature rule is to find a set of points $x_{i}$ and weights $w_{i}$ that approximates the integral $I\left(T\right)$ by a weighted sum of function evaluations. According to the spherical-radial cubature rule [30], the Equation (22) can be rearranged as:

(23) $$I\left(T\right)\approx \overset{m}{\underset{i=0}{\sum }}w_{i}T\left(\xi_{i}\right)$$

where $w_{i}=1/m,i=1,2\ldots \ldots \ldots ,m,m=2n$. $\xi_{i}$ is the cubature point located at the intersection of the unit sphere and its axes, $\xi_{i}=\sqrt{\frac{m}{2}}{\left[1\right]}_{i}$, $\left[1\right]=\left\{\left(\begin{array}{l} 1\\ .\\ 0 \end{array}\right),\ldots ,\left(\begin{array}{l} 0\\ .\\ 1 \end{array}\right),\ldots ,\left(\begin{array}{l} -1\\ .\\ 0 \end{array}\right),\ldots ,\left(\begin{array}{l} 0\\ .\\ -1 \end{array}\right)\right\}$.

#### 3.2.2. Cubature Kalman Filter Algorithm Process

In the time update process, the Bayesian filter computes the mean ${\hat{x}}_{k|k-1}$ and the associated covariance $P_{k|k-1}$ of the Gaussian predictive density.

Assume at time k that the posterior density function is known. The cubature points $X_{i,k-1|k-1}$ can be calculated as:

(24) $$X_{i,k-1|k-1}=\sqrt{P_{k-1|k-1}}\xi_{i}+{\hat{x}}_{k-1|k-1}$$

where $P_{k-1|k-1}$ is the error covariance matrix at time instant k − 1; $\xi_{i}$ is Basic cubature points.

The transmission of cubature points can be evaluated as the following:

(25) $$X_{i,k|k-1}^{*}=F_{k-1}X_{i,k|k-1}$$

where $F_{k-1}$ is the known matrix function.

The state prediction ${\hat{x}}_{k|k-1}$ and the covariance matrix of state prediction $P_{k|k-1}$ can be achieved as follows:

(26) $${\hat{x}}_{k|k-1}=\frac{1}{m}\overset{m}{\underset{i=1}{\sum }}X_{i,k|k-1}^{*}$$

(27) $$P_{k|k-1}=\frac{1}{m}\overset{m}{\underset{i=1}{\sum }}X_{i,k|k-1}^{*}X_{i,k|k-1}^{*T}-{\hat{x}}_{k|k-1}{\hat{x}}_{k|k-1}^{T}+Q_{k-1}$$

where $Q_{k-1}$ is the system noise covariance.

It is well known that errors in the predicted measurements are zero-mean white sequences. Under the assumption that these errors can be well approximated by the Gaussian, the cubature points $X_{i,k|k-1}$ can be evaluated (i = 1, 2………, m, m = 2n):

(28) $$X_{i,k|k-1}=\sqrt{P_{k|k-1}}\xi_{i}+{\hat{x}}_{k|k-1}$$

Then the transmission of cubature points $Z_{i,k|k-1}$ can be obtained:

(29) $$Z_{i,k|k-1}=h\left(X_{i,k|k-1},v_{k}\right)$$

where $h\left(.\right)$ is known function, $v_{k}$ is the measurement noise.

Then measurement prediction ${\hat{z}}_{k|k-1}$ can be described as:

(30) $${\hat{z}}_{k|k-1}=\frac{1}{m}\overset{m}{\underset{I=1}{\sum }}Z_{i,k|k-1}$$

Combining Equations (29) and (30), the innovation covariance matrix $P_{zz,k|k-1}$ can be estimated:

(31) $$P_{zz,k|k-1}=\frac{1}{m}\overset{m}{\underset{I=1}{\sum }}Z_{i,k|k-1}Z_{i,k|k-1}^{T}-{\hat{z}}_{k|k-1}{\hat{z}}_{k|k-1}^{T}+R_{k}$$

where $R_{k}$ is the measurement noise covariance.

The cross-covariance matrix $P_{xz,k|k-1}$ can be calculated:

(32) $$P_{xz,k|k-1}=\frac{1}{m}\overset{m}{\underset{I=1}{\sum }}X_{i,k|k-1}Z_{i,k|k-1}^{T}-{\hat{x}}_{k|k-1}z_{k|k-1}^{T}$$

Combining Equations (31) and (32), The Kalman gain $W_{k}$ can be described:

(33) $$W_{k}=P_{zz,k|k-1}P_{xz,k|k-1}^{-1}$$

The state update ${\hat{x}}_{k|k}$ can be written as:

(34) $${\hat{x}}_{k|k}={\hat{x}}_{k|k-1}+W_{k}\left(z_{k}-{\hat{z}}_{k|k-1}\right)$$

Since the state needs to be normalized further, the state update ${\hat{x}}_{k|k}$ can be computed as the following:

(35) $${\hat{x}}_{k|k}={\hat{x}}_{k|k}/\left\|{\hat{x}}_{k|k}\right\|$$

The corresponding error covariance $P_{k|k}$ can be estimated as:

(36) $$P_{k|k}=P_{k|k-1}-W_{k}P_{zz,k|k-1}W_{k}^{-1}$$

### 3.3. Adaptive Cubature Kalman Filter Algorithm

**Figure 4:**
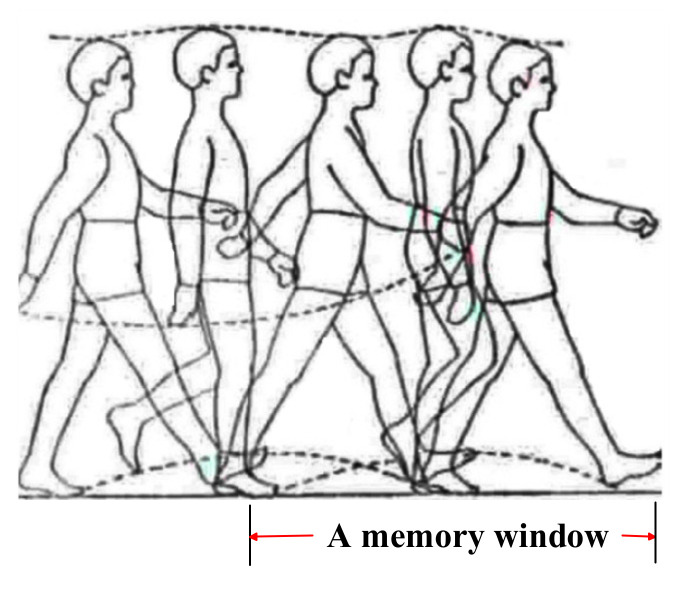
A memory window of the limited memory weighted method.

The traditional cubature Kalman filter algorithm requires the statistical characteristics of the system state noise and measurement noise. In practical applications, due to the complexity of the environment, it is difficult to obtain statistics of noises, which introduces uncertainties and causes the prediction accuracy to decrease or even diverge [30,43]. To dynamically correct the system noise and measurement noise, the Sage-Husa estimator has been applied to the CKF algorithm to update the noise covariance estimator, ${\hat{Q}}_{k}$ and ${\hat{R}}_{k}$. The Sage-Husa filter algorithm applies to the non-Gaussian noise case. If dynamic noise and observation noise is non-correlated, the algorithm estimates the noise variance by a fading factor, continuously adjusting the system model in a recursive algorithm to modify model parameters confirmed by prior information [44,45]. Since the Sage-Husa filter algorithm uses the method of average information distribution, the contribution of noise parameters to the estimation is 1/k. However, the sensors of the smartphone are low cost and inaccurate. The noise parameters of MARG sensors are easily changed by the external environment. Therefore, the noise parameters updated in real-time can better reflect the current data characteristics. It is necessary to emphasize the role of the latest measurement information, and gradually weaken the effect of stale information. To estimate the noise parameters more accurately, the noise covariance was estimated using the fading memory weighted method and the limited memory weighted method in this paper. According to the characteristics of pedestrian walking, the latest step data was used as a memory window in the pedestrian walking process, as depicted in Figure 4. Since the limited memory weighted method requires the covariance of the estimated and predicted values at the k-w moment to be known, the paper used the fading memory weighted method to calculate the model noise parameters from the starting time to the k-w moment. Then from the moment k − w + 1, the noise covariance was calculated by the limited memory weighted method. This paper estimated and corrected the model noise parameters by combining a fading memory weighted method and limited memory weighted method to improve the accuracy of filter estimation.

In general, the statistical properties of general nonlinear systems were considered as follows:

(37) $$\left\{\begin{array}{c} E\left(\omega_{k}\right)=q\;\;\;\;cov\left(\omega_{k},\omega_{j}^{T}\right)=Q\delta_{kj}\\ E\left(v_{k}\right)=r\;\;\;\;cov\left(v_{k},v_{j}^{T}\right)=R\delta_{kj}\\ cov\left(\omega_{k},v_{j}^{T}\right)=0; \end{array}\right.$$

where q and r are the means of system noise and measurement noise respectively, Q is the covariance matrix of system noise, and R is the covariance matrix of measurement noise.

In the fading memory weighted method, the weighted factor $\lambda_{i}$ can be rewritten as:

(38) $$\lambda_{i}=\lambda_{i-1}b,0.95<b<0.99,\overset{k}{\underset{i=1}{\sum }}\lambda_{i}=1$$

where $\lambda_{i}=d_{k}b^{i-1}$, $d_{k}=(1-b)/(1-b^{k+1})$, i = 1, 2……k, b is the forgetting factor.

For real-time measurement noise, it is necessary to add parameter value λ to determine the filter memory length. The smaller the value of λ, the greater effect of the latest observations on the current estimate.

According to the Sage-Husa maximum posterior estimation algorithm and time-variant noise statistic estimator, the measurement noise covariance ${\hat{R}}_{k}$ and the state noise covariance ${\hat{Q}}_{k}$ of the fading memory weighted method can be expressed as follows [31]:

(39) $${\hat{R}}_{k}=\left(1-d_{k}\right){\hat{R}}_{k-1}+d_{k}[\varepsilon_{k}\varepsilon_{k}^{T}-(\frac{1}{2n}\overset{m}{\underset{I=1}{\sum }}Z_{i,k|k-1}Z_{i,k|k-1}^{T}-{\hat{z}}_{k|k-1}z_{k|k-1}^{T})]$$

(40) $${\hat{Q}}_{k}=\left(1-d_{k}\right){\hat{Q}}_{k-1}+d_{k}[K_{k}\varepsilon_{k}\varepsilon_{k}^{T}K_{k}^{T}+P_{k|k}-(\frac{1}{2n}\overset{m}{\underset{i=1}{\sum }}X_{i,k|k-1}^{*}X_{i,k|k-1}^{*T}-{\hat{x}}_{k|k-1}{\hat{x}}_{k|k-1}^{T})]$$

where $\varepsilon_{k}$ is the filter innovation, and $\varepsilon_{k}=z_{k}-{\hat{z}}_{k|k-1}$.

When the movement state of the system changes rapidly, the latest observation data is particularly important for estimating the noise statistics at the current time, and the effect of the old data is small [46,47]. Due to the dynamic of smartphone sensor noise, the estimated value of noise statistics at the current time has a stronger correlation with the latest historical data. To increase the weight of the latest historical data, a limited memory weighted method was used to calculate the estimate of the noise parameter. The limited memory weighted method is an exponential weighting method for fixed-length historical data before the current time.

This paper proposed that the latest step data is selected as the length of the memory window in the pedestrian walking process, as shown in Figure 4. In the limited memory weighted method, the weighted factor β_i_ can be rewritten as:

(41) $$\beta_{i}=\beta_{i-1}b;\;0.95<b<0.99,\overset{k}{\underset{i=1}{\sum }}\beta_{i}=1$$

where $\beta_{i}=d_{w}b^{i-1}$, $d_{w}=\left(1-b\right)/\left(1-b^{w}\right)$, and b is the forgetting factor. After the k-w moment, replace the weight coefficients in Equations (38)–(39) with $\beta_{k+1-i}$, then in the limited memory adaptive filter the measurement noise covariance ${\hat{R}}_{k}$ and the state noise covariance ${\hat{Q}}_{k}$ can be expressed as:

(42) $${\hat{R}}_{k}=b{\hat{R}}_{k-1}+d_{w}[\varepsilon_{k}\varepsilon_{k}^{T}-(\frac{1}{2n}\overset{2n}{\underset{i=1}{\sum }}Z_{i,k|k-1}Z_{i,k|k-1}^{T}-{\hat{z}}_{k|k-1}z_{k|k-1}^{T})]+d_{w}b^{w}{\hat{R}}_{k-w}$$

where

$${\hat{R}}_{k-w}=\varepsilon_{k-w}\varepsilon_{k-w}^{T}-(\frac{1}{2n}\overset{2n}{\underset{i=1}{\sum }}Z_{i,k-w|k-w-1}Z_{i,k-w|k-w-1}^{T}-{\hat{z}}_{k-w|k-w-1}z_{k-w|k-w-1}^{T})$$

(43) $${\hat{Q}}_{k}=b{\hat{Q}}_{k-1}+d_{w}[K_{k}\varepsilon_{k}\varepsilon_{k}^{T}K_{k}^{T}+P_{k|k}-(\frac{1}{2n}\overset{2n}{\underset{i=1}{\sum }}X_{i,k|k-1}^{*}X_{i,k|k-1}^{*T}-{\hat{x}}_{k|k-1}{\hat{x}}_{k|k-1}^{T})]+d_{w}b^{w}{\hat{Q}}_{k-w}$$

where

$${\hat{Q}}_{k-w}=W_{k-w}\varepsilon_{k-w}\varepsilon_{k-w}^{T}W_{k-w}^{T}+P_{k-w|k-w}-(\frac{1}{2n}\overset{2n}{\underset{i=1}{\sum }}X_{i,k-w|k-w-1}^{*}X_{i,k-w|k-w-1}^{*T}-{\hat{x}}_{k-w|k-w-1}{\hat{x}}_{k-w|k-w-1}^{T})$$

By analyzing the recursive process of the filter, it is found that if the Kalman gain becomes smaller and smaller, the role of the new measurement data reflecting the true state to a certain extent is getting weaker and weaker in the estimation, which may result in data saturation and causes the filter to diverge. Therefore, the main measure to restrain the filter divergence is to pay attention to the role of new measurement data in the current filter. In practical applications, when filtering divergence occurs, the measurement noise covariance R and the state noise covariance Q always lose semi-positive or positive definiteness, and then the filter variance will diverge. For Equations (39), (40), (42) and (43), we found that when the absolute values of the non-diagonal non-zero elements of the cross-covariance on the right side of the Equations (39), (40), (42) and (43) were so great to a certain degree, or there were negative values in the diagonal elements of the error covariance, it will make R and Q lose their positive definiteness. Therefore the loss of positive semidefiniteness of Q and the loss of positive definiteness of R can be regarded as a sign of filter divergence. As long as Q and R are always positive semidefinite and positive definite during the recursive calculation process, the filtering divergence can be prevented.

In this paper, based on the biased noise variance estimation method, the measurement noise covariance R and the state noise covariance Q were corrected by the filter innovation for restraining the divergence when Q loses positive semidefiniteness and R loses positive definite. For the fading memory weighted method, the corrected measurement and state noise covariance are as follows:

(44) $${\hat{R}}_{k}=\left(1-d_{k}\right){\hat{R}}_{k-1}+d_{k}\varepsilon_{k}\varepsilon_{k}^{T}$$

(45) $${\hat{Q}}_{k}=\left(1-d_{k}\right){\hat{Q}}_{k-1}+d_{k}K_{k}\varepsilon_{k}\varepsilon_{k}^{T}K_{k}^{T}$$

For the limited memory weighted method, the corrected measurement and state noise covariance can be expressed as follows:

(46) $${\hat{R}}_{k}=b{\hat{R}}_{k-1}+d_{w}\varepsilon_{k}\varepsilon_{k}^{T}+d_{w}b^{w}{\hat{R}}_{k-w}$$

(47) $${\hat{Q}}_{k}=b{\hat{Q}}_{k-1}+d_{w}K_{k}\varepsilon_{k}\varepsilon_{k}^{T}K_{k}^{T}+d_{w}b^{w}{\hat{Q}}_{k-w}$$

In actual circumstances, the moving object is generally difficult to maintain regular motion. So, it is very difficult to construct an accurate functional model. Moreover, during the movement of the carrier, the carrier will inevitably be affected by abnormal interference from the outside world, resulting in the state model not being able to truly reflect the movement law of the carrier. To overcome the filter model error and the influence of abnormal disturbance, an adaptive factor (α) is applied by using predicted state discrepancy statistics to overcome the abnormal influence of state disturbance [39]. In this paper, the adaptive factor is the two-segment function together with the statistic of the predicted state discrepancy, which can be represented as:

(48) $$\partial_{k}=\left\{\begin{array}{cc} 1, & \Delta {\tilde{V}}_{k}\leq c0\\ \frac{c0}{\Delta {\tilde{V}}_{k}}, & \Delta {\tilde{V}}_{k}>c0 \end{array}\right.$$

where c0 is a constant, which can be adjusted depending on the practical implementation, usually c0 = 2.0 − 3.0.; $\Delta {\tilde{V}}_{k}$ is the statistic of the predicted state discrepancy, defined as $\Delta {\tilde{V}}_{k}={\left[\varepsilon_{k}^{T}\varepsilon /tr\left({\hat{P}}_{k|k-1}\right)\right]}^{\frac{1}{2}}$.

To control the influence of the dynamic model error, the adaptive factor is applied for correcting the Kalman gain. Having weakened the negative impacts of measurement outliers and state model errors, the innovation covariance matrix $P_{zz,k|k-1}^{*}$ and the cross-covariance matrix $P_{xz,k|k-1}$ can be expressed as:

(49) $$P_{zz,k|k-1}^{*}=\frac{1}{\partial_{k}}(\frac{1}{m}\overset{m}{\underset{I=1}{\sum }}Z_{i,k|k-1}Z_{i,k|k-1}^{T}-{\hat{z}}_{k|k-1}{\hat{z}}_{k|k-1}^{T})+R_{k}$$

(50) $$P_{xz,k|k-1}=\frac{1}{\partial_{k}}(\frac{1}{m}\overset{m}{\underset{I=1}{\sum }}X_{i,k|k-1}Z_{i,k|k-1}^{T}-{\hat{x}}_{k|k-1}z_{k|k-1}^{T})$$

## 4. Experiments and Result Analysis

To evaluate the proposed approach, we conducted extensive experiments in both static and dynamic situations. The smartphone MI 5 was selected as the test device, which was embedded with MARG sensors. As for comparisons, EKF, CKF, and SHCKF were used as baselines. The initial state noise and measurement noise covariance matrices of the proposed filter were empirically determined depending on each measurement outputted by the smartphone in the static and dynamic test [25]. For static tests, $Q=diag(e^{-5},e^{-5},e^{-5})$, $R=diag(e^{-3},e^{-3},e^{-3})$, and for the dynamic test, $Q=diag(e^{-4},e^{-4},e^{-4})$, $R=diag(e^{-3},e^{-3},e^{-3})$, where $diag\left(.\right)$ represents a diagonal matrix. Moreover, in static and dynamic tests the sampling frequency of data was 50 Hz. The forgetting factor b and the constant c0 were empirically determined, and the values for b and *c*0 were 0.96 and 2.1 respectively.

### 4.1. Experiment in the Static Condition and Result Analysis

**Figure 5:**
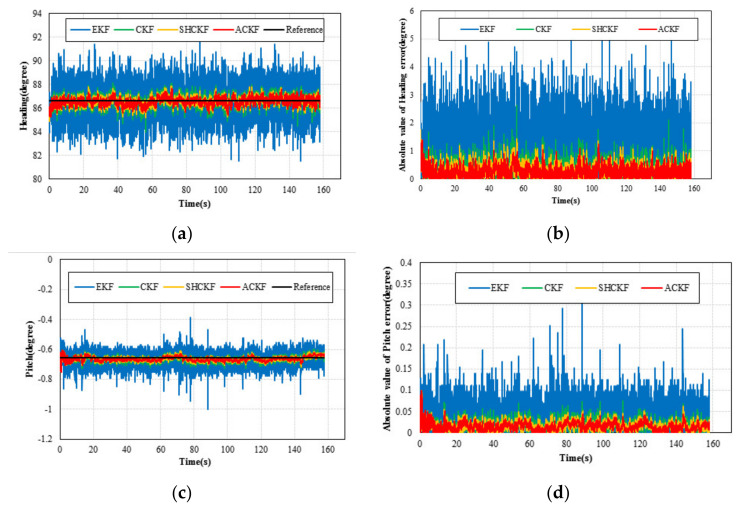

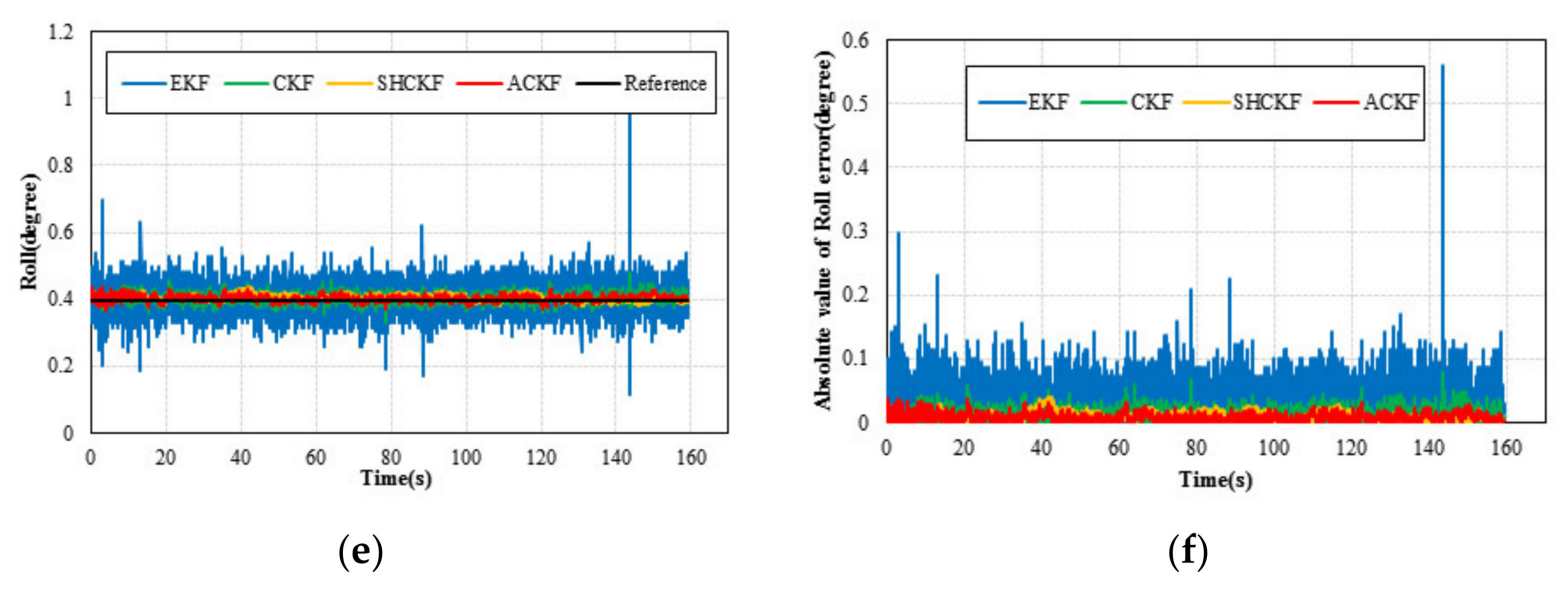
The results of the static test: (**a**) heading angle; (**b**) absolute value of the heading error; (**c**) pitch angle; (**d**) absolute value of pitch error; (**e**) roll angle; and (**f**) absolute value of the roll error.

**Figure 6:**
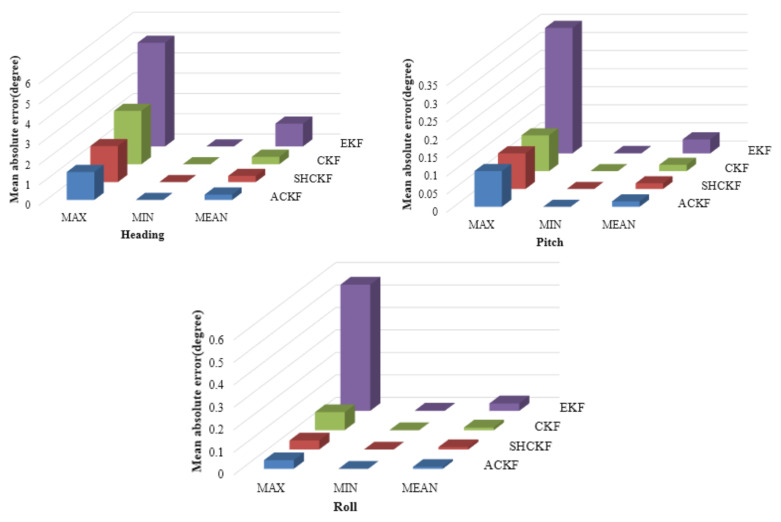
Distributions of mean absolute errors of extended Kalman filter (EKF), cubature Kalman filter (CKF), Sage-Husa cubature Kalman filter (SHCKF), and ACKF.

In the static test, a MI 5 smartphone was placed on the desktop to collect MARG sensors data. Since the smartphone was stationary, a sampling period was selected as the length of the memory window. Although the attitude and heading values calculated from the outputs of the accelerometer and magnetometer fluctuated severely, the average of a sample set with enough size could be used as a reference [25]. Figure 5 shows the absolute error and angle of the attitude and heading of the adaptive cubature Kalman filter (ACKF), Sage-Husa cubature Kalman filter (SHCKF), cubature Kalman filter (CKF), and extended Kalman filter (EKF) results in the static test. From Figure 5, we can see that the absolute value of the attitude and heading errors of the EKF results were larger and more unstable than those of the CKF, SHCKF, and ACKF results. This is because the EKF algorithm uses linearization to approximate nonlinear functions, so its estimation accuracy is not high. The CKF, SHCKF, and ACKF algorithms employ a third-degree spherical-radical cubature rule to compute the Gaussian-weighted integrals numerically and use cubature point sets to approximate the mean and variance. So, CKF, SHCKF, and ACKF have stronger adaptability than EKF. Besides, although the results of ACKF, SHCKF, and CKF were similar, the mean absolute error (MAE) of the ACKF results was best in Table 1. Since the ACKF method emphasized the role of the latest measurement information, and gradually weakened the effect of stale information. The noise covariance was estimated using the fading memory weighted method and the limited memory weighted method to estimate the noise parameters more accurately. Table 1 and Figure 6 present the error absolute value of the ACKF, SHCKF, CKF, and EKF algorithms results. According to the results of Table 1, the MAE of ACKF results were more accurate than those of SHCKF, CKF, and EKF algorithms. Compared with the SHCKF, CKF, and EKF, the mean absolute error (MAE) of the heading of the ACKF results decreased to about 13.21%, 28.70%, and 76.55% respectively. Moreover, the ACKF reduced to about 4.46%, 14.77%, and 61.24% of the MAE of pitch compared to the other algorithms respectively. Additionally, the MAE of the roll of the ACKF results diminished to about 17.14%, 28.69%, and 73.95% respectively.

### 4.2. Experiment in the Dynamic Condition and Result Analysis

**Figure 7:**
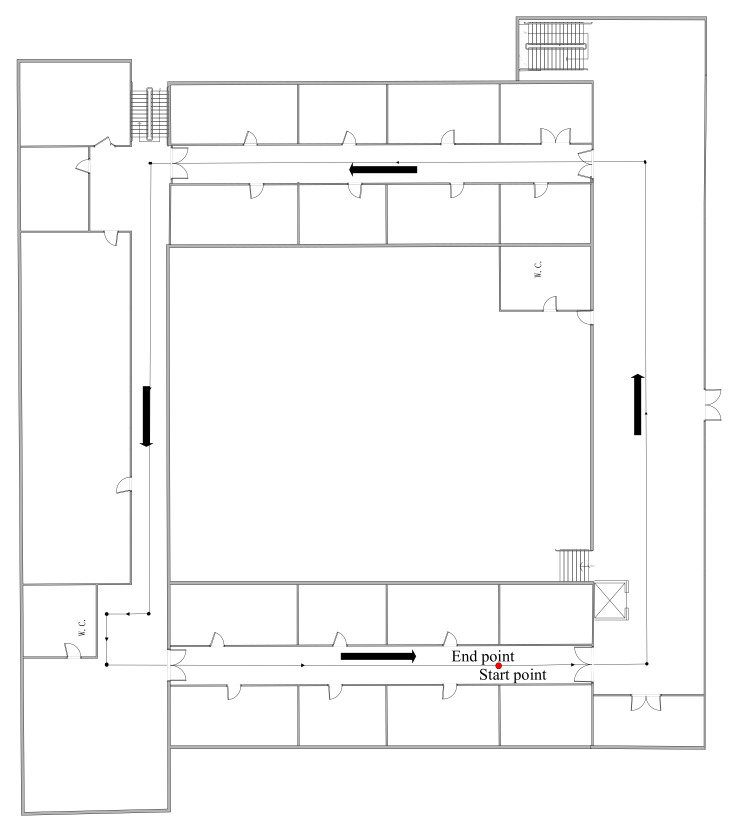
Floor plan of the site for the dynamic test at a corridor.

To further verify the superiority of the proposed ACKF on attitude and heading estimation, the dynamic test was conducted in the corridors on the first floors of a research building. The floor plans are presented in Figure 7. In the dynamic test, we held the MI 5 smartphone at a constant speed through a corridor, as shown in Figure 6. During the experiment, there were sudden turns, and the movement state of the smartphone was changed during the sudden turn. Due to the reference values of roll and pitch cannot be measured, the dynamic test focused on processing and comparing heading results. Due to the reference heading being difficult to achieve in a dynamic test, the estimation error cannot be directly presented. This paper used two methods to assess the heading error. On the one hand, the dynamic test was conducted in the corridor. As the start point and the end point were the same and in the middle of a straight corridor, the average heading of a straight corridor at the beginning can be used as a reference value for the heading at the end. On the other hand, the location tracking performance of PDR can reflect heading estimation performance to some extent [25]. Generally, research on PDR includes three aspects: heading estimation, location tracking, and speed estimation. Heading estimation is the focus of this article. Location tracking is based on the primary theory of dead reckoning [25,36]. The speed estimation research mainly includes stride detection and step length estimation. For stride detection, it can be obtained by calculating the measured total acceleration peak. Besides an empirical model is employed for step length estimation as the following. Detailed implementation refers to [25,36].

(51) $$StepLength=S\times \sqrt[{4}]{A_{max}-A_{min}}$$

where $A_{max}$ and $A_{min}$ are the maximum and minimum vertical acceleration in a single step; S is the personalized parameter that needs to be calibrated for each pedestrian.

Moreover, the roll and pitch information from the smartphone potentially improves the heading estimation, because it also considers the effects of the roll and pitch [24]. The measured values of an accelerometer, magnetometer, and gyroscope in the body coordinate system can be converted to the horizontal plane of the navigation coordinate system:

(52) $$\begin{array}{l} a^{l}=C_{b}^{l}a_{b}=C_{b}^{l}{\left[a_{x}\;a_{y}\;a_{z}\right]}^{T}\\ m^{l}=C_{b}^{l}m_{b}=C_{b}^{l}{\left[m_{x}\;m_{y}\;m_{z}\right]}^{T}\\ w^{l}=C_{b}^{l}w_{b}=C_{b}^{l}{\left[w_{x}\;w_{y}\;w_{z}\right]}^{T} \end{array}$$

where $a^{l}=\left[a_{x}^{l}\;a_{y}^{l}\;a_{z}^{l}\right]$ is the projection of accelerometer measurements in the horizontal plane, $m^{l}=\left[m_{x}^{l}\;m_{y}^{l}\;m_{z}^{l}\right]$ is the projection of magnetometer measurements in the horizontal plane, and $w^{l}=\left[w_{x}^{l}\;w_{y}^{l}\;w_{z}^{l}\right]$ is the projection of gyroscope measurements in the horizontal plane.

Combined with the step length and heading of the current step, the two steps of the relative displacement increment are given as:

(53) $$\begin{array}{l} X_{k+1}=X_{k}+StepLength\times cos(\psi )\\ Y_{k+1}=Y_{k}+StepLength\times sin(\psi ) \end{array}$$

where *x* and *y* represent the *x*-axis and *y*-axis values in the n-frame, and k + 1 and k represent the respective step counts; StepLength is the step length; and ψ is the heading in the n-frame.

In the dynamic test, we used the location tracking performance of PDR to reflect heading estimation performance. There were three people involved in the experiment. Each participant had different heights and weights, as shown in Table 2. The length of the corridor that they walked was as long as 148.39 m each.

**Figure 8:**
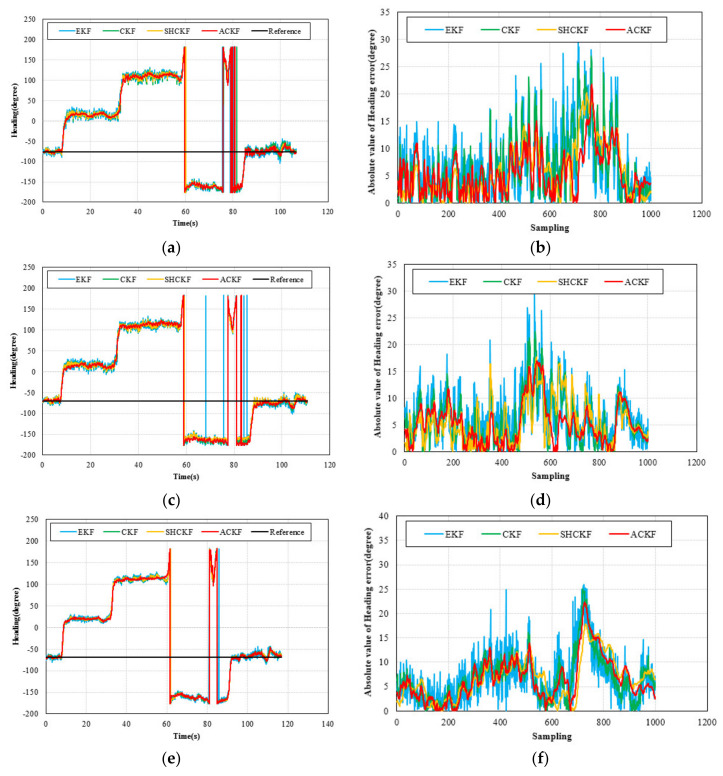
Distributions of heading and the absolute value of heading error for three participants, (**a**,**b**) participant 1, (**c**,**d**) participant 2, and (**e**,**f**) participant 3.

**Figure 9:**
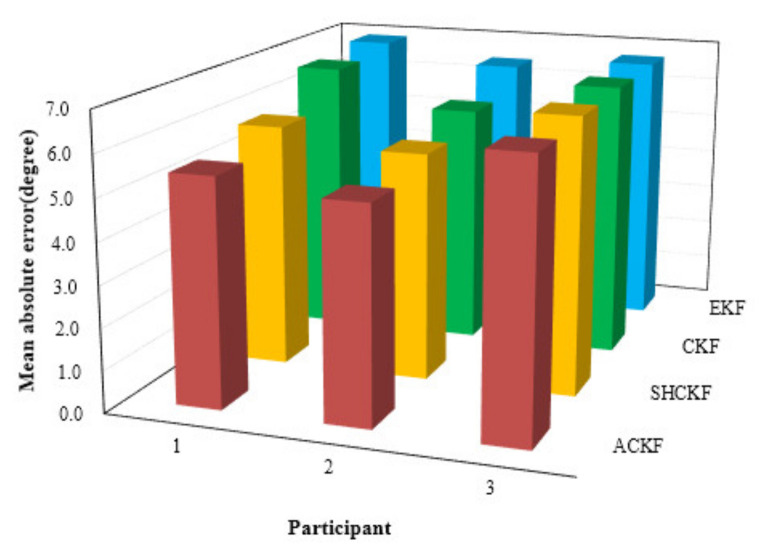
Distributions of the MAE of EKF, CKF, SHCKF, and ACKF results with respect to three participants.

The heading results in the dynamic test are presented in Figure 8. Figure 8a,c,e shows the results of the heading. The black line in Figure 8 is the heading reference from the initial heading. Figure 8b,d,f presents the absolute value of heading error for the last twenty seconds of the test. From Figure 8, we can see that the proposed ACKF algorithm could provide a more stable and accurate heading estimation information compared with the EKF, CKF, and SHCKF algorithms. Table 3 and Figure 9 give the statistical results of the heading error. As we can see from Table 3, the mean absolute error (MAE) of the heading of the ACKF results reduced about 7.10%, 18.61%, and 20.69% respectively in the first participant test. The MAE of the heading for the second participant test decreased about 5.93%, 11.72%, and 19.68% respectively. In addition the last participant results decreased 1.69%, 2.28%, and 2.58% respectively. Due to the complex and changeable indoor environment, there were multiple interference sources. When pedestrians are walking with their smartphones in their hands, there is still swaying and shaking, which also have a certain impact on the heading. Therefore, the real-time heading angle calculated by the smartphone sensor not only includes the actual heading angle of the pedestrian but also may include deviations caused by environmental influences and pedestrian swaying and shaking. From the heading results of the dynamic experiment, it is found that the improvement of the proposed algorithm accuracy is not obvious. This can be explained that in the dynamic experiment, due to the complex indoor environment, there are multiple interference sources. When pedestrians are walking with their smartphones in their hands, there is still swaying and shaking, which also have a certain impact on the heading. Therefore, the real-time heading angle calculated by the smartphone sensor not only includes the actual heading angle of the pedestrian but also may include deviations caused by environmental influences and pedestrian swaying and shaking. The pedestrian swaying and shaking may cause fluctuations in heading results at many points and interfered with the average heading. Meanwhile, the reference heading angle used in this paper was the average value of the heading at the initial stage, which can only reflect the average value of heading changes to a certain extent, and cannot reflect the filtering performance well. Therefore, the accuracy improvement was not obvious. Besides, heading experiment results only verified the last 1000 sampling data, and the data accuracy of the whole dynamic experiment was not compared. So, the PDR location tracking method was proposed to verify the accuracy of whole dynamic experiments.

**Figure 10:**
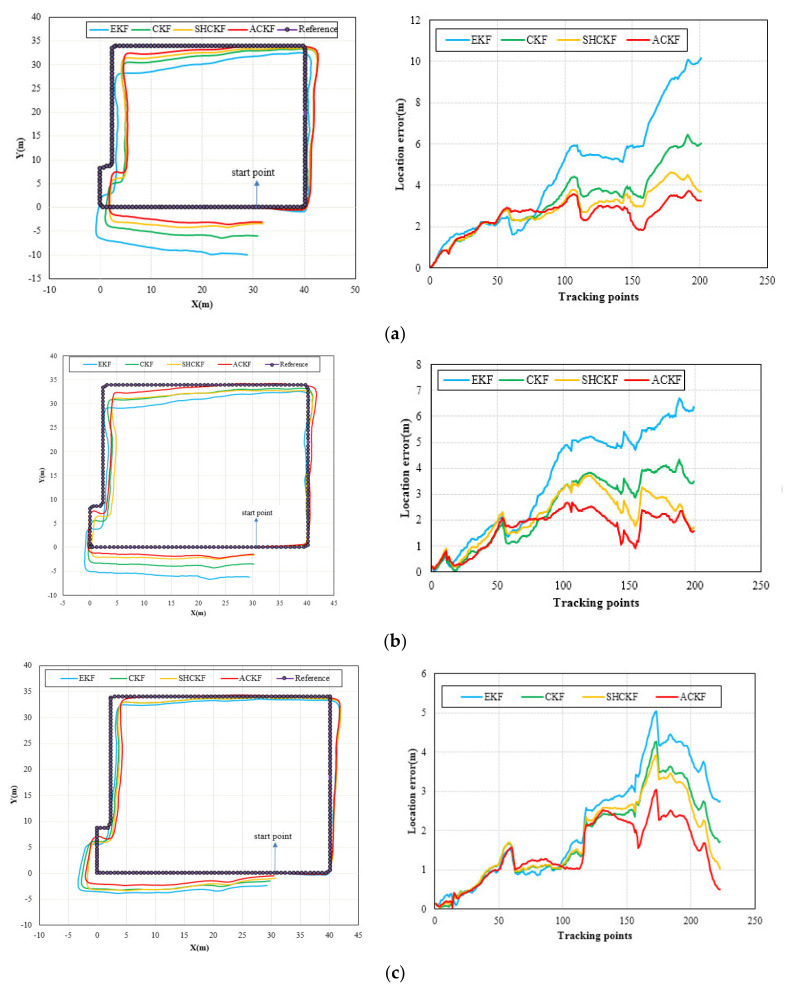
Distributions of location errors in location tracking of EKF, CKF, SHCKF, and ACKF for three participants, (**a**) participant 1, (**b**) participant 2, and (**c**) participant 3.

**Figure 11:**
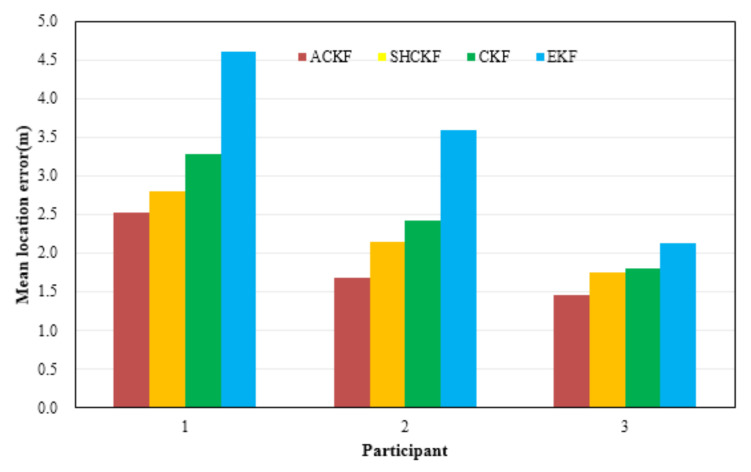
Distributions of the mean of location errors in location tracking for three participants.

Figure 10 shows the results of location tracking, which can reflect more intuitive improvements of heading estimation. The black line in Figure 10 is the reference trace. Figure 10 illustrates the comparison of the location and location errors calculated by the ACKF, SHCKF, CKF, and EKF algorithms. In Figure 10, for all of the three participants, compared with the results of EKF, the results of the CKF, SHCKF, and ACKF approximate the reference trace better for the three participants. Since the EKF uses the first-order approximation to approximate nonlinear functions, which will accumulate errors and decrease the estimation accuracy. Moreover, the results of SHCKF and ACKF were accurate and stable than those of CKF. This is due to ACKF and SHCKF algorithms introducing the optimal adaptive factor so that they can accurately track the uncertainty of the model errors. Besides, the accuracy of the ACKF results was the best. Since the ACKF algorithm uses the fading memory weighted method and the limited memory weighted method to estimate the noise parameters, which can emphasize the role of the latest measurement information, and gradually weakens the effect of stale information, improving the estimation accuracy. Moreover, an adaptive factor is applied to overcome the abnormal influence of sudden turns. All three figures indicate that ACKF, SHCKF, CKF, and EKF provide low location errors at the beginning of tracking. However, ACKF performs and the walking distance becomes longer. Experimental results show that there was a problem of error accumulation in PDR, and ACKF could better solve this problem to a certain extent. Table 4 and Figure 11 give the statistical results of the location errors. Compared with the SHCKF, CKF, and EKF, the mean of location errors of the ACKF results decreased about 9.92%, 23.24%, and 45.33% respectively in the first participant test. The mean of location errors of the second participant ACKF results decreased by 21.62%, 30.51%, 53.13%, and the last participant results decreased by 17.36%, 19.80%, and 32.06%.

In general, the results of the static test show that the proposed ACKF could provide optimal models for attitude and heading estimation. In the location tracking test, it is noticeable that the proposed ACKF had smaller errors and was more stable compared with the SHCKF, CKF, and EKF. Meanwhile, for PDR, the statistical characteristics of pedestrian moving were dynamically changing. The proposed ACKF filter could adapt to dynamic conditions. Therefore, it could be concluded that the proposed ACKF method could achieve better accuracy making it more suitable for indoor positioning.

## 5. Discussion

**Figure 12:**
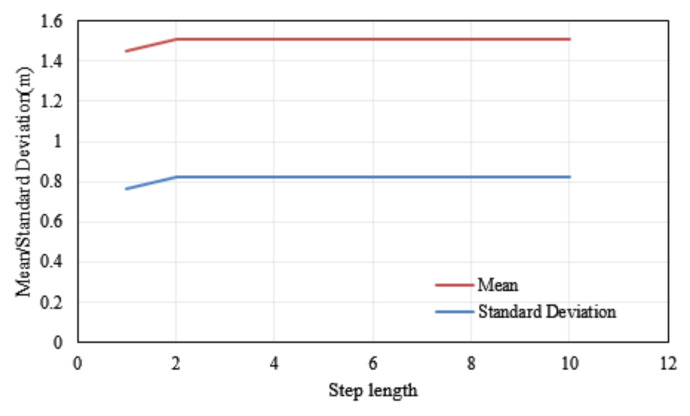
Distributions of the mean and standard deviation different steps as the memory window.

This paper proposed a quaternion-based adaptive cubature Kalman filter (ACKF) to estimate the attitude and heading of a smartphone with its embedded sensors’ data. According to the characteristics of pedestrian walking, this paper proposed that the latest step data was used as the memory window data of the limited memory weighted method. In the process of pedestrian walking, the state of the pedestrian was usually stable within one step, and the output position in the pedestrian dead reckoning algorithm was separated by steps. At the same time, we found that the closest step data had the strongest correlation with the current heading through experimental comparison. Figure 12 presents the mean and standard deviation of the latest 1–10 step data as the memory window. As we can see from Figure 12, when the latest step data was selected as the length of the memory window, the mean and standard deviation of the results was the smallest.

**Figure 13:**
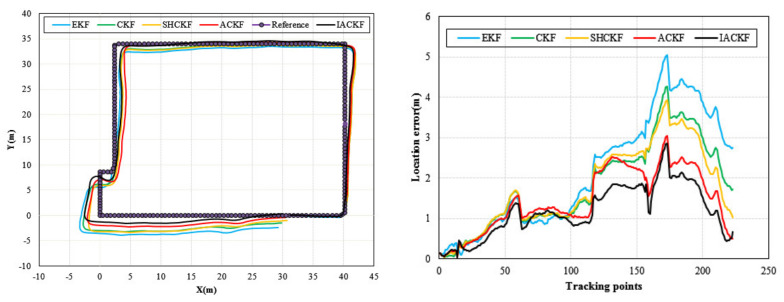
Distributions of different methods.

To evaluate the proposed approach, we conducted extensive experiments in both static and dynamic situations. In the static experiment, the proposed algorithm outperformed the other three filters remarkably, while in the dynamic test, the superiority of the proposed one over the others was not so great. The reason is that the complex environment and changeable conditions, such as swaying and shaking of the pedestrian’s body during the dynamic test could cause severe fluctuations in heading results. Thus, the attitude information from the MARG sensors potentially improved the heading estimation by considering the effects of the roll and pitch [24]. Considering the influence of attitude, we derived headings in the horizontal plain. Take an experiment participant as an example, the results are presented in Figure 13. From Figure 13, we can see that the improved ACKF algorithm (IACKF) results were closest to the actual path compared with the other methods. Table 5 gives the statistical results of the location errors. The mean and standard deviation of the results obtained by the improved algorithm was the smallest.

Due to the complex indoor environment, there were multiple sources influencing attitude and heading estimation. The proposed algorithm could alleviate the negative impact of the inaccurate setting of the noise covariance matrix to a certain extent, but the position error could be still accumulated, so adaptive and robust algorithms with better performances need to be investigated in the future. Considering the randomness of the way users hold smartphones, we will consider identifying more complex pedestrian activities in the next step.

## 6. Conclusions

This paper proposed a quaternion-based adaptive cubature Kalman filter algorithm for attitude and heading estimation fused with the outputs of MARG sensors. The fading memory weighted method and the limited memory weighted method were used to reduce the weight of stale data and adaptively modify the model noise parameters. The filter innovation sequence was used to rectify the measurement noise covariance and system noise covariance matrices for restraining the divergence. Besides the adaptive factor is applied by using predicted state discrepancy statistics to overcome the sudden steering of state disturbance. The static and dynamic experiments were conducted in an indoor environment to verify the superiority of the proposed algorithm. In terms of the heading, the proposed algorithm could provide a more stable and accurate heading estimation information. For the attitude, the proposed algorithm could effectively improve the accuracy under quasistatic condition. Moreover, in the dynamic test, the heading calculated by EKF, CKF, SHCKF, and ACKF was input into the PDR method respectively. The location tracking performance shows that the heading calculated by the proposed algorithm could make the location estimation more accurate.

## Figures and Tables

**Table 1:** Statistical results of the absolute value of heading, pitch, and roll error in the static test.

	Error Metrics	ACKF	SHCKF	CKF	EKF
The absolute value of Heading error (degree)	Max	1.3793	1.7745	2.6325	5.1126
	Min	0.0000	0.0000	0.0000	0.0001
	Mean	0.2641	0.3043	0.3704	1.1261
The absolute value of Pitch error (degree)	Max	0.0989	0.0984	0.0990	0.3469
	Min	0.0000	0.0000	0.0000	0.0010
	Mean	0.0150	0.0157	0.0176	0.0387
The absolute value of Roll error (degree)	Max	0.0385	0.0403	0.0802	0.5616
	Min	0.0000	0.0000	0.0000	0.0018
	Mean	0.0087	0.0105	0.0122	0.0334

**Table 2:** Detailed information of all participants (S is the step length parameter).

Participant	Sex	Height (m)	Weight (kg)	S
1	Male	1.75	87	0.48
2	Female	1.72	80	0.48
3	Male	173	80	0.46

**Table 3:** Comparisons of the mean absolute error (MAE) of heading (degree).

Participant	Error Metrics	ACKF	SHCKF	CKF	EKF
First	Mean	5.4628	5.8802	6.7118	6.8883
Second	Mean	5.1625	5.4881	5.8480	6.4277
Third	Mean	6.5167	6.6284	6.6687	6.6890

**Table 4:** Comparisons of mean localization errors (m).

Participant	Error Metrics	ACKF	SHCKF	CKF	EKF
First	Mean	2.5227	2.8005	3.2866	4.6148
Second	Mean	1.6805	2.1441	2.4185	3.5855
Third	Mean	1.4508	1.7556	1.8089	2.1353

**Table 5:** Comparisons of the mean and standard deviation of different methods.

Error Metrics	IACKF	ACKF	SHCKF	CKF	EKF
Mean (m)	1.1813	1.4508	1.7556	1.8089	2.1353
Standard Deviation (m)	0.6382	0.7646	1.0282	1.0900	1.4251

## Data Availability

Data sharing is not applicable to this article.
